# Viral Shedding and Persistence of Anosmia and Ageusia in an Asymptomatic SARS-CoV-2 Infection

**DOI:** 10.7759/cureus.36574

**Published:** 2023-03-23

**Authors:** Nikolaos Kintrilis

**Affiliations:** 1 Internal Medicine, National and Kapodistrian University of Athens, Athens, GRC

**Keywords:** long-covid, viral shedding, taste loss, smell loss, ageusia, anosmia, covid-19, sars-cov-2

## Abstract

The novel severe acute respiratory syndrome coronavirus 2 (SARS-CoV-2), first identified in the region of Wuhan, China is responsible for the ongoing pandemic of coronavirus disease-19 (COVID-19) that has been a part of our life for almost three years now. Although there have been multiple reports of prolonged viral shedding in people with severe disease, viral shedding lasting for extended periods can occur in patients with less serious clinical insults or even asymptomatic individuals. Herein, we report a case of a female patient that, although otherwise asymptomatic, remained positive on nasopharyngeal viral testing for a prolonged period, alongside persisting complaints of anosmia and ageusia. The patient may well have been one of the first individuals to be infected in the Greek territory; we followed up on her long-term COVID sequelae from the time of proof of infection up until the present day.

## Introduction

The severe acute respiratory syndrome coronavirus2 (SARS-CoV-2) novel coronavirus was the cause of the ongoing pandemic that has had a worldwide effect for almost three years now. It was first identified in Wuhan, China in late 2019 [1]. The first case of coronavirus disease-19 (COVID-19) in Greece was reported on February 26, 2020, and since then, more than 5.18 million cases have been recorded which have cost the lives of 33,750 people [2]. The disease can present with a plethora of clinical manifestations, ranging from asymptomatic infection to the more common viral syndrome and even severe acute respiratory distress syndrome leading to multiple complications and death [3]. Viral shedding refers to the period during which a SARS-CoV-2-infected patient tests with a positive nasopharyngeal swab, sputum sample, or endotracheal aspirate sample, with detection of the virus, usually being quantified by real-time reverse-transcriptase polymerase chain reaction (RT-PCR). Even though viral shedding times do not seem to differ significantly based on the severity of the disease, a more severe course of the disease commonly predicts prolonged viral shedding [4]. Long-term health complications of COVID-19, commonly termed long-COVID, post-COVID state, or post-COVID syndrome have been the object of multiple large studies. Complications of the disease include a plethora of clinical and psychosocial manifestations, while the management of the syndrome has evolved as a challenge for national health systems and rehabilitation services worldwide [5-7].

In the present study, we report the case of a patient with a mild infection who presented with prolonged viral RNA shedding alongside anosmia and ageusia; we also discuss the patient’s follow-up.

## Case presentation

A 50-year-old female nurse presented at the emergency department of a Greek third-level hospital on February 12, 2020 with complaints of loss of smell and taste for the past few days. No further symptomatology was mentioned by the patient and she denied the presence of any fever, cough, disordered breathing, or any other lung manifestations. Clinical examination showed all vital signs were within normal limits and did not reveal any pathological findings. A full laboratory profiling was made, the results of which are presented in Table 1. A chest X-ray was ordered which also proved normal. An otorhinolaryngology consult was made which revealed no apparent findings. The patient mentioned no history of medical conditions and was receiving no drugs at the time. She denied any alcohol, tobacco, or recreational drug use. The patient had not travelled recently, however, she mentioned close contact with a friend of her daughter who had recently returned from a trip to Italy. A nasopharyngeal swab was obtained and RT-PCR for SARS-CoV-2 was performed, which was positive.

**Table 1:** Laboratory panel of the patient upon presentation to the emergency room.

Parameter	Patient value	Reference range
Haematology		
White blood cells (K/μL)	6.3	4.0-10.8
Red blood cells {M/μL)	4.1 (low)	4.2-5.6
Haemoglobin (g/dL)	13.2	12.0-16.0
Haematocrit (%)	37.5 (low)	38.0-46.0
Mean corpuscular volume (fL)	91.2	80.0-97.0
Platelets (K/μL)	270	150-440
Biochemistry		
Sodium (mmol/L)	141	135-150
Potassium (mmol/d)	4.7	3.6-5.0
Calcium (mg/dL)	9.7	8.1-10.5
Total bilirubin (mg/dL)	0.70	0.20-1.20
Total protein (g/dL)	6.7	6.0-8.3
Albumin (g/dL)	4.2	3.5-5.0
Lactate dehydrogenase (U/L)	259	200-460
Creatinophosphokinase (U/L)	236 (high)	26-145
Aspartate aminotransferase (U/L)	15	9-36
Alanine aminotransferase (U/L)	16	10-28
γ-glutamyltransferase (U/L)	20	9-30
Alkaline phosphatase (U/L)	103 (high)	33-98
Low-density lipoproteins (mg/dL)	103	0-160
High-density lipoproteins (mg/dL)	61	42-88
Triglycerides (mg/dL)	72	35-135
Uric acid (mg/dL)	3.6	2.6-6.0
Glucose (mg/dL)	94	70-110
Urea (mg/dL)	42	10-50
Creatinine (mg/dL)	0.8	0.6-1.3
Ferritin (mg/L)	46.1	10.0-150.0
Thyroid stimulating hormone (mIU/L)	2.50	0.30-5.00
Vitamin D (ng/mL)	24 (low)	30-100
Imaging		
Chest X-ray	normal	

The patient was advised strict at-home isolation and daily evaluation of her general status, temperature, and pulse oximetry values. She was instructed to record the onset of any new symptoms and, since she was one of our first confirmed cases, we kept in daily communication with her via telephone. During the next fourteen days, the period for which her initial quarantine lasted, her condition remained absolutely stable without the occurrence of any new symptoms. On the fourteenth day, the patient returned to the hospital for a second PCR test which proved positive, while at the same time, she mentioned the persistence of olfactory and taste dysfunction. We decided to advise in favour of a quarantine extension of seven more days and re-evaluate the patient. Upon her return on the 21st day, both anosmia and ageusia persisted, while the patient remained otherwise asymptomatic; she also tested with a positive PCR test for the third time. Based on the Greek National Public Health Organization’s guidelines from the time, the patient’s quarantine was ended and she was able to return to the community and her job. We continued follow-up on a weekly basis with repeated PCR testing and clinical and laboratory examinations.

Positive PCR testing persisted over the next weeks until on May 6, 2020, twelve weeks after her initial presentation, the patient tested negative for SARS-CoV-2. A final nasopharyngeal specimen was acquired for PCR testing one week later which was again negative, confirming successful eradication of the virus from the patient’s oropharynx. During these follow-up weeks, the patient remained perfectly healthy showing no signs of fatigue or other new symptoms, and being able to carry out her job duties and daily activities as before. Of note, her laboratory values showed no derailments whatsoever during the observation time. However, since the loss of smell and taste were of unchanged severity according to the patient’s report, we opted for a brain MRI which revealed mild thickening of the paranasal sinuses as well as scoliosis of the nasal septum, while the brain parenchyma was without pathological findings. No further action was deemed necessary at the time.

**Figure 1:**
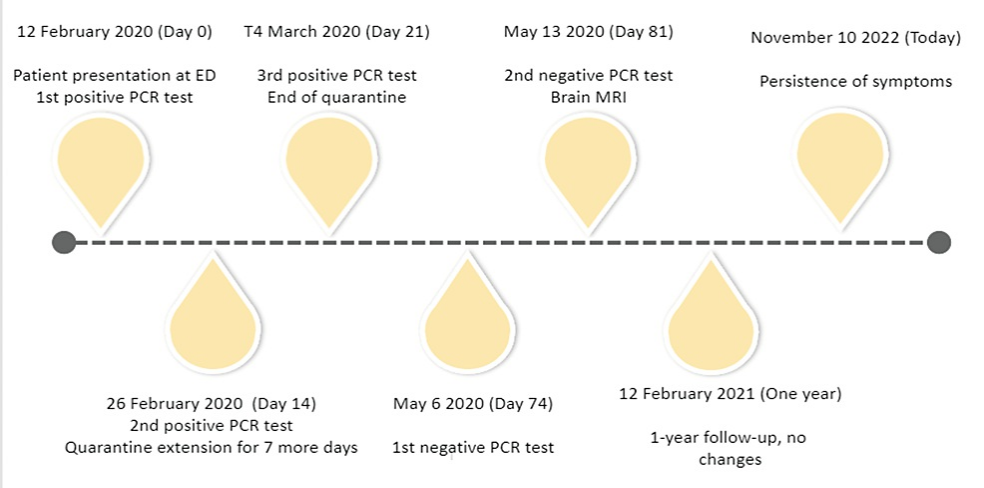
Main timestamps during the follow-up of the patient so far. PCR: polymerase chain reaction.

For the next period, we followed-up with the patient on a monthly basis for clinical evaluation of her persisting symptoms. Today, almost three years later, the patient is in perfect health but with the symptoms of anosmia and ageusia still affecting her everyday life. The main events during the period for which the patient was observed are depicted in Figure 1. Although no objective tests have been performed so far, the patient still complains of complete lack of smell and taste, having been more or less accustomed to these.

## Discussion

According to reports from all over the globe, SARS-CoV-2-affected individuals are slowly but surely declining in most countries, as herd immunity is forming. However, the new concept of COVID-19 long-term sequelae, collectively termed ‘long-COVID’ or ‘post-COVID condition’ arises more in the current medical literature. Olfactory disorders as a complaint in post-COVID patients have been described in many patients following SARS-CoV-2 infection recovery, but most patients seem to recover olfaction sooner or later, usually by one year after the diagnosis [8]. Though the exact pathophysiological mechanism remains under investigation, it has been hypothesized that decreased sensitivity of sensory neurons and competitive activity of the virus on ACE2 receptors pertain to the mechanisms involved [9-10]. Prolonged viral shedding of the novel coronavirus has been observed in COVID-19 patients with risk factors including symptom severity, advanced age, female sex, obesity, and time from symptom onset to hospital admission or commencement of treatment [11-13].

Herein, we describe the case of a female patient that exhibited prolonged SARS-CoV-2 viral shedding of approximately 11 weeks, as well as extremely long-persisting anosmia and ageusia, lasting since the beginning of the pandemic until today. The patient was otherwise in perfect health and was able to return fully to her job and everyday tasks without further post-COVID manifestations whatsoever. We consider this an interesting added proof of how even asymptomatic or mildly symptomatic COVID-19 patients may exhibit long-term clinical manifestations of the disease that can impact their everyday life. Further research is required to explore various post-COVID manifestations pathophysiology in an effort to collectively put an end to the pandemic. 

## Conclusions

Our case demonstrates a rare case of extremely prolonged viral shedding which may represent a large viral load of the respiratory tract - although the patient showed no apparent symptomatology from the respiratory system, as well as persisting changes in sense of smell and taste lasting up to the present day. We consider this an interesting group of individuals that could be further studied to add to the knowledge around long-COVID manifestations.

## References

[1] Zhu N, Zhang D, Wang W (2020). A novel coronavirus from patients with pneumonia in China, 2019. N Engl J Med.

[2] (2022). Worldometers coronavirus cases. https://www.worldometers.info/coronavirus/.

[3] Esakandari H, Nabi-Afjadi M, Fakkari-Afjadi J, Farahmandian N, Miresmaeili SM, Bahreini E (2020). A comprehensive review of COVID-19 characteristics. Biol Proced Online.

[4] Munker D, Osterman A, Stubbe H (2021). Dynamics of SARS-CoV-2 shedding in the respiratory tract depends on the severity of disease in COVID-19 patients. Eur Respir J.

[5] Koc HC, Xiao J, Liu W, Li Y, Chen G (2022). Long COVID and its management. Int J Biol Sci.

[6] Crook H, Raza S, Nowell J, Young M, Edison P (2021). Long covid-mechanisms, risk factors, and management. BMJ.

[7] Subramanian A, Nirantharakumar K, Hughes S (2022). Symptoms and risk factors for long COVID in non-hospitalized adults. Nat Med.

[8] Renaud M, Thibault C, Le Normand F, Mcdonald EG, Gallix B, Debry C, Venkatasamy A (2021). Clinical outcomes for patients with anosmia 1 year after COVID-19 diagnosis. JAMA Netw Open.

[9] de Melo GD, Lazarini F, Levallois S (2021). COVID-19-related anosmia is associated with viral persistence and inflammation in human olfactory epithelium and brain infection in hamsters. Sci Transl Med.

[10] Park JW, Wang X, Xu RH (2022). Revealing the mystery of persistent smell loss in long COVID patients. Int J Biol Sci.

[11] Long H, Zhao J, Zeng HL (2021). Prolonged viral shedding of SARS-CoV-2 and related factors in symptomatic COVID-19 patients: a prospective study. BMC Infect Dis.

[12] Zhang S, Zhu H, Ye H (2021). Risk factors for prolonged virus shedding of respiratory tract and fecal in adults with severe acute respiratory syndrome coronavirus-2 infection. J Clin Lab Anal.

[13] Owusu D, Pomeroy MA, Lewis NM (2021). Persistent SARS-CoV-2 RNA shedding without evidence of infectiousness: a cohort study of individuals with COVID-19. J Infect Dis.

